# Voltage-Gated K^+^ Channel Modulation by Marine Toxins: Pharmacological Innovations and Therapeutic Opportunities

**DOI:** 10.3390/md22080350

**Published:** 2024-07-29

**Authors:** Rita Turcio, Francesca Di Matteo, Ilaria Capolupo, Tania Ciaglia, Simona Musella, Carla Di Chio, Claudio Stagno, Pietro Campiglia, Alessia Bertamino, Carmine Ostacolo

**Affiliations:** 1 Department of Pharmacy, University of Salerno, 84084 Fisciano, Italy; rturcio@unisa.it (R.T.); fdimatteo@unisa.it (F.D.M.); icapolupo@unisa.it (I.C.); tciaglia@unisa.it (T.C.); smusella@unisa.it (S.M.); pcampiglia@unisa.it (P.C.); abertamino@unisa.it (A.B.); 2 Department of Chemical, Biological, Pharmaceutical and Environmental Sciences (CHIBIOFARAM), University of Messina, 98166 Messina, Italy; carla.dichio@unime.it (C.D.C.);

**Keywords:** voltage-gated potassium channels, marine toxins, peptides, disease treating

## Abstract

Bioactive compounds are abundant in animals originating from marine ecosystems. Ion channels, which include sodium, potassium, calcium, and chloride, together with their numerous variants and subtypes, are the primary molecular targets of the latter. Based on their cellular targets, these venom compounds show a range of potencies and selectivity and may have some therapeutic properties. Due to their potential as medications to treat a range of (human) diseases, including pain, autoimmune disorders, and neurological diseases, marine molecules have been the focus of several studies over the last ten years. The aim of this review is on the various facets of marine (or marine-derived) molecules, ranging from structural characterization and discovery to pharmacology, culminating in the development of some “novel” candidate chemotherapeutic drugs that target potassium channels.

## 1. Introduction

### 1.1. Voltage-Gated Ion Channels

Different species from the three primary super kingdoms of life use ion flow, a signaling mechanism that is mediated by ion channels activation across excitable cells, extensively. Ion channels produce and organize electrical signals that control several vital physiological functions, including the heartbeat, thought processes in the brain, and muscular contraction. Throughout the biological world, the ion channels are membrane proteins that form small, water-filled pores, along with their electrochemical gradients, to mediate high rates and selective flux of ions, enabling diverse physiological functions in cells [1]. Complex proteins, called voltage-gated ion channels, are anchored in the lipid membrane of cells. The voltage across the membrane controls the extraordinarily high rates of ion conductance (~1 million ions per second) in these channels [2]. Ion-selective pores guide a particular class of ions (Na^+^, K^+^, Ca^2+^, and Cl^−^) as a response to different stimuli that induce the opening and closing of the pores, in a mechanism called “gating”. Thus, ion channels can be grouped into families according to their ion selectivity and mode of gating. For example, voltage-gated K^+^ channels are activated by the depolarization of the membrane potential, resulting in conformational changes that allow K^+^ ions to permeate. Channels that are activated by voltage and are selective for potassium ions are called K_v_ channels; likewise, those selective for sodium and calcium channels are called Na_v_ and Ca_v_, respectively [3].

**Figure 1:**
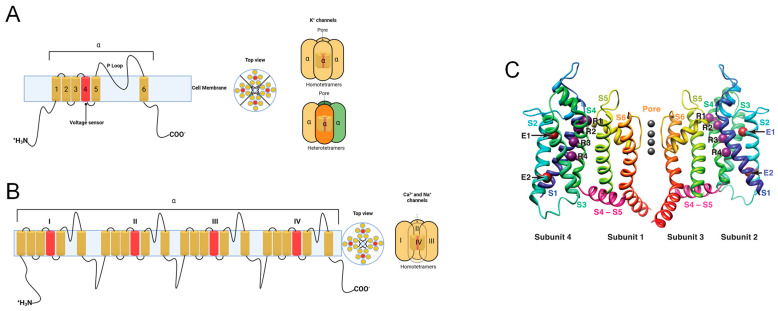
Schematic representation of the proposed transmembrane topology of voltage-gated ion channels. (**A**) The α-subunit of K^+^ channels showing the transmembrane segments (1–6) spanning the cell membrane. Segment 4 (red color) represents the voltage sensor. In the extracellular region, the S5-S6 P-loop is directly involved in the ion conduction pathway. In the intracellular region, the N and C termini ends of the polypeptide appear. The right panel shows the top and side views of homo- or heterotetramers of encircled four-fold α-subunits forming the central ion conduction pathway of a K^+^ channel. (**B**) A single subunit polypeptide of four homologous domains (I–IV) forming a functional pore of Ca^2+^ and Na^+^ channels. (**C**) K_v_1.2 channel structural models in activated and resting states X-ray crystallography were used to identify the structure of the K_v_1.2 channel in an activated state, and the ROSETTA membrane technique was used to predict the channel’s structure in a closed state. The voltage-sensing and pore-forming modules consist of two subunits. Take note of the labels that show how the voltage-sensing module of subunit 4 connects with the subunit 1 pore-forming module (left) and how the subunit 2 voltage-sensing module interacts with the subunit 3 pore-forming module (right). S1, dark blue; S2, light blue-green; S3, light green; S4, dark green; S5, yellow-green; and S6, orange, are the colored transmembrane segments. The S4–S5 linkers covalently connecting pore-forming and voltage-sensing modules are highlighted in magenta [7].

Many genes encoding ion channels have been cloned, expressed, and described using techniques such as molecular biology, electrophysiology, and structural biology. Several studies have demonstrated their structural similarity [2]. The channel’s architecture has been now clearly outlined. The primary pore-forming α-subunit of voltage-gated ion channels (Figure 1) is functionally composed of four different α-subunits (K^+^ channels) or a single α-subunit with four homologous domains (Na^+^ and Ca^2+^ channels). According to the literature [4,5,6], the latter could be a homomeric or heteromeric tetramer. Six hydrophobic transmembrane segments, S1–S6, make up the α-subunit. The S5 and S6 segments are connected by the P-loop across the extracellular side of the membrane. The P-loop plays a direct role in the creation of the selectivity filter and the conductance pathway. Modulatory effects are produced by the α-subunits’ interactions with auxiliary subunits.

**Figure 2:**
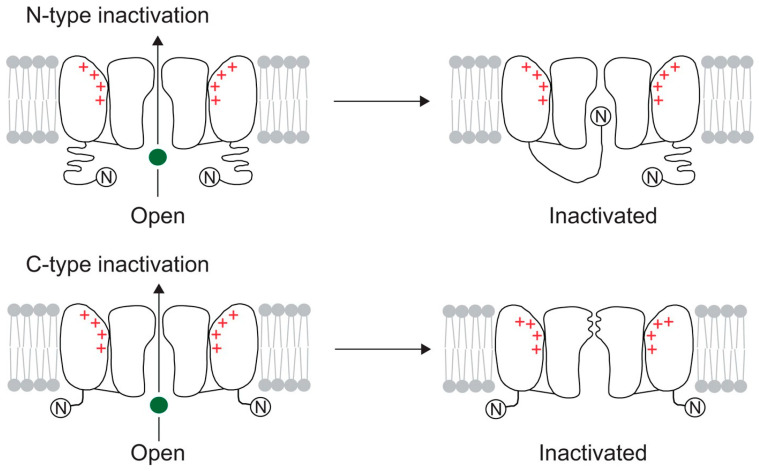
According to the “ball and chain” model of inactivation, the ion channels contain a domain (“ball”) tethered to the cytoplasmatic side of the protein. Following a conformational change, the inactivation ball is free to bind to its receptor, occluding the ion-conducting pore. This fast inactivation process (N-type inactivation) can be distinguished for K^+^ channels from a second inactivation process (C-type inactivation), which involves a conformational change in the extracellular part of the protein. The K^+^ is depicted as a green circle [12].

To regulate ion permeation, the structure of voltage-gated ion channels must be equipped with a charged transmembrane domain suitable for detecting membrane potential changes. The voltage sensor has been identified as a series of highly conserved, positively charged amino acids in the S4 transmembrane segment. Upon depolarization, the sensor reacts by displacement of these charged residues, resulting in a set of conformational changes in the protein. Finally, this gating process leads to the pore opening and ion conduction through the channel [8]. When the membrane is depolarized, some voltage-gated ion channels first open and then enter a non-conducting, ‘inactivated’ state. This inactivation process influences some key signaling properties of excitable cells, such as action-potential duration, shape, and firing frequency. At least two different mechanisms—rapid N-type or gradual C-type—can cause inactivation (Figure 2). When the internal mouth of a K^+^ channel opens, a region close to the amino terminus is involved in N-type inactivation, which results in the formation of a tethered inactivation particle [9].

The rapid inactivation of ion channels has been explained by the “ball and chain” model mechanism (Figure 2). Fast inactivation of ion channels is dependent on a hydrophobic motif of triplet residues (IFM) in the III-IV linker. It has been proposed that the IFM motif acts as a “latch” to keep the fast inactivation gate closed. Synthetic peptides were, initially, used for investigating the main chemical features of the ball domain required for potassium channel inactivation. “Ball” peptides deriving from one K^+^ channel can also inactivate other K^+^ channels, thus suggesting that the ball-and-chain inactivation has low specificity and needs a correct hydro-lipophilic balance in the aminoacid sequence [10,11]. On the other hand, specificity of “chain” peptides in N-type inactivation kinetics remains questionable. Recently, it has been postulated that an adequate chain should contain a minimum of 12 residue spacers between the transmembrane region and the triplet motif, located at the N terminus, which appears to be essential for inactivation [10]. Unlike N-type inactivation, the primary factor in C-type inactivation is probably pore reconfiguration rather than movement of a channel’s cytoplasmic portion. 

Cryo-EM studies demonstrate that the C-type inactivation of K_v_1 channels involves the dilation of the external pore due to displacement of the P-loop [12,13], while the C-type inactivation of K_v_2 channels involves dynamic alterations in electromechanical coupling that reposition the pore-lining S6 helices and closes the internal pore [14]. 

### 1.2. Voltage-Gated K^+^ Channels (VGKCs)

Voltage-gated K^+^ channels (K_v_) are tetrameric plasma membrane proteins mediating the selective flow of K^+^ ions down their electrochemical gradient in response to a depolarization in the transmembrane electric field. Compared to other ion channel families, the K_v_ channel family is more widespread and diversified [15]: it is made up of 40 family members, organized in 12 different subfamilies [16], expressed in excitable and non-excitable tissues of organisms, from bacteria to humans [17]. In non-excitable cells, K^+^ channels play an important role in cell proliferation, cell volume regulation and lymphocyte differentiation [18], while, in excitable tissues, they are involved in diverse physiological functions, such as setting the membrane resting potential and controlling the shape and frequency of action potentials [9,12]. The selectivity and voltage dependence of K_v_ channels make them central players in several physiological functions, including the maintenance and modulation of neuronal [19] and muscular (both cardiac and skeletal) excitability [17], the regulation of calcium signaling cascades, hormonal secretion, and others [20]. 

Structurally, K_V_ channels are composed of four separate α-subunits, each containing six transmembrane segments (S1–S6) [21]. The S1 and S4 segments make the voltage sensing domain (VSD), which controls the pore opening via the S4-S5 intracellular loop that is connected to the pore domain (PD). The PD is formed by the transmembrane segments S5–S6, including a re-entrant pore loop bearing the potassium selectivity sequence [T/S]VG[Y/F]G [22]. More specifically, the peptide chain (H5 or loop P), between the segments S5 and S6, lines the water-filled channel pore. This region is highly conserved in all voltage-gated ion channels. Mutations in this region alter the permeation properties of the ion channel. K_V_ channels have an S4 segment, bearing a cluster of positively charged residues (e.g., lysines and arginines), which serves as the voltage sensor. Voltage-dependent fast inactivation (N-type) of the channel is mediated by the “ball and chain” mechanism. Several residues in S6 are involved in the slow inactivation process (C-type) [4].

Mammalian voltage-gated potassium channels were originally classified into four major subfamilies based on their closeness to *Drosophila*-related genes: *Shaker* (K_v_1), *Shab* (K_v_2), *Shaw* (K_v_3), and *Shal* (K_v_4). Neurons can generate an immense variation in potassium channel characteristics by hetero-multimerizing various subtypes generated from the same channel subfamily [23]. Mutations in the K_v_ channel gene cause physiological and electrical alterations; that is why K_v_ channels have been linked to several significant diseases: heart arrhythmias (such as long-QT syndromes), congenital deafness, epilepsy, diabetes, blood pressure dysregulation, multiple sclerosis, Alzheimer’s disease and other dementias, migraine, pain, anxiety, and bipolar disorder are a few of them [24,25]. Given their physiological significance, K_v_ channels are targets for several small molecules and natural toxins. These ion channels are closely monitored as possible targets for drugs to treat many different diseases, since the malfunctioning of these VGICs is linked to several major illnesses [26,27,28].

## 2. K_v_ Channel Blockers from Molluscan Peptides

Molluscan peptides have become an important resource of molecules with pharmacological activity, displaying a wide range of bioactivities, such as antibacterial, analgesic, and neuroactive properties. Most of these noteworthy effects are mediated by modulation of voltage-gated potassium channels (K_v_) and all their subtypes, which are important regulators of immunological responses, muscular contraction, neuronal communication, and other physiological processes. This is why understanding the structural and functional aspects of these peptides and their interactions with K_v_ channels holds promise for the development of novel therapeutics targeting neurological disorders, autoimmune diseases, and other pathological conditions [29]. The discovery and identification of physiologically active compounds produced by marine venomous species can drive the advancement of scientific disciplines, including pharmacology, medicine, and molecular and evolutionary biology [30].

### 2.1. Conotoxin

**Figure 3:**
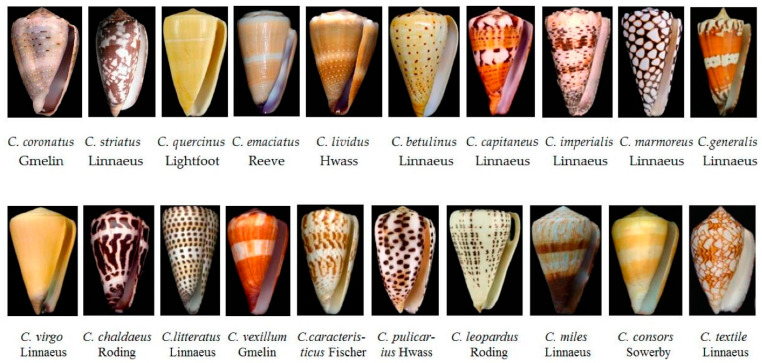
Twenty of the most abundant *Conus* species in the South China Sea [34].

An outstanding variety of invertebrate species may be found in coral reef environments, and many of them employ bioactive substances as part of their defense or prey-capture tactics. In these diverse habitats, the cone snail, belonging to the genus *Conus*, from the family *Conidae* (Figure 3), which consists of over ~600 predatory species of mollusks, [31] has proven to be a rich source of peptide toxins, also known as conopeptides or conotoxins. The toxin peptides are produced for prey immobilization and defense against predators, to deter competitors, and for other biological purposes [32,33].

**Figure 4:**
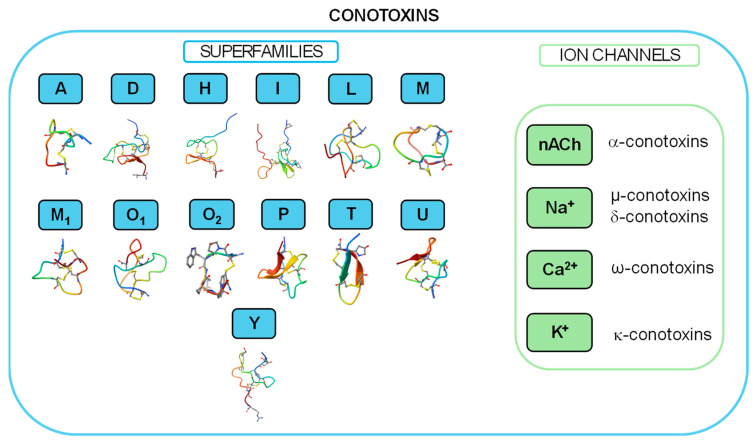
A structural schematic illustration to show the classification of conotoxins into superfamilies and their ion channel targets.

Currently, conotoxins are divided into major superfamilies, as shown in Figure 4. According to the cysteine organization, each superfamily can be further subdivided into numerous subfamilies. Moreover, according to their different selectivity and affinity for ion channels [35], conotoxins are further classified as α-conotoxins, which target the nicotinic acetylcholine receptor [36]; µ-conotoxins and δ-conotoxins, modulating Na^+^ channels [37]; ω-conotoxins, specifically blocking N-type Ca^2+^ channels and exhibiting high affinity for certain subtypes of Ca^2+^ channels [38]; and κ-conotoxins, targeting K^+^ channels [39,40].

**Figure 5:**
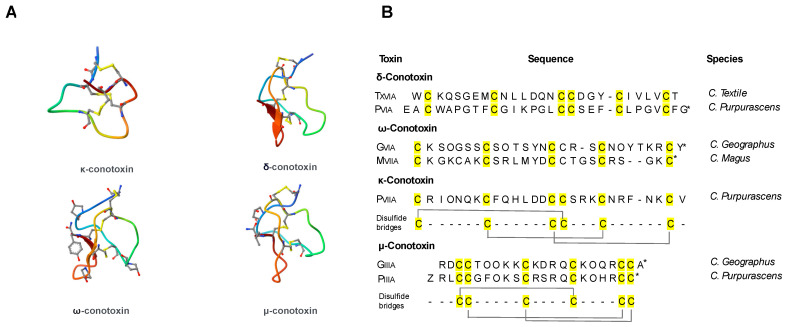
Primary structure of conotoxins binding to voltage-gated ion channels. (**A**) µ- and δ-conotoxins (2EFZ, 1FUE) interact with Na^+^ channels, ω- with Ca^2+^ channels, and κ- with K^+^ channels (1TTL, 1KCP). K^+^ channel peptide blockers seem to possess a common functionally important dyad consisting of a hydrophobic residue and key lysine protruding from a relatively flat surface [45]. (**B**) These residues are highlighted in bold in the κ-PVIIA primary sequence [41,46].

Conopeptides’ range size is typically between 8 and 30 amino acids in length, in contrast with the average size of toxin polypeptides from other venoms, which are typically composed of 40–80 amino acids [41]. According to their cysteine composition, the *Conus* peptide population shows a big diversity in their structure, as result of differences in disulfide bond numbers and frameworks [42,43,44]. Residue substitutions at critical positions in the sequence resulted in the formation of the required toxin surface for highly potent and selective interactions with their receptors (Figure 5). Conotoxins serve as crucial tools for understanding various biological processes, given their capacity to differentiate between distinct receptor subtypes. Cone snails have a remarkable molecular specificity in developing ligands that are directed towards voltage- and ligand-gated ion channel superfamilies [43].

Considering the nature of this review, we will focus on ĸ-contoxins, which inhibit K^+^ channels. *Conus* peptides have been used to define specific molecular forms within a large ion channel family and to investigate the functional biology of a particular ion channel subtype. This specificity, displayed by conopeptides, makes them of exceptional interest and has led to their widespread use as research tools in neurobiology. Table 1 summarizes the main conotoxin potencies and/or efficacies for the different K^+^-channel targets.

#### 2.1.1. κ-Conotoxin PVIIA

**Figure 6:**
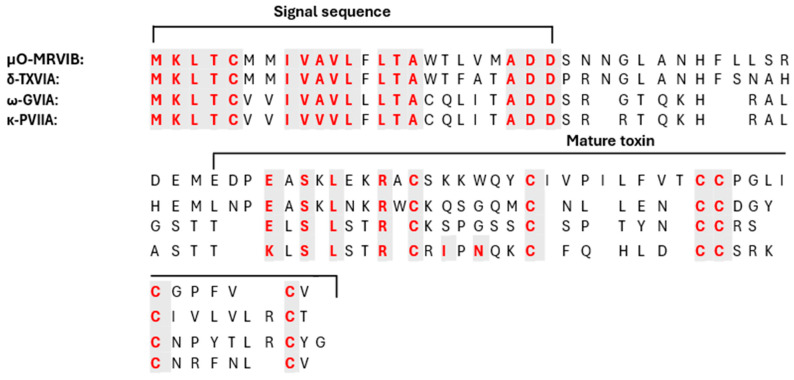
A comparison of O-superfamily precursor sequences. The conserved cysteine pattern is illustrated in bold. The inferred sequence of the μO-conotoxin MrVIB precursor sequence is aligned with the prepropeptide sequences of Δ-conotoxin TxVIA (formerly called the King-Kong peptide), ω-conotoxin GVIA, and к-PVIIA. The conserved amino acids are illustrated with a grey background.

κ-Conotoxin PVIIA (κ-PVIIA), found in the fish-hunting snail *C. purpurascens*, is the first conotoxin identified with activity over K^+^ channels, representing the first identified member of the O-superfamily, with a length of 27–34 amino acids, containing six cysteine residues combined to generate three or four disulfide bonds (Figure 6) [39]. It has been demonstrated that this conopeptide binds with high affinity to the voltage-gated *Shaker* K^+^ channel at nM concentrations [40]. This structural class marine toxin has been shown to inhibit K^+^ conductance in oocytes containing the cloned K^+^ channel derived from *Drosophila’s* *Shaker* [40,41,60,61], binding to the external vestibule in a voltage-sensitive manner and blocking channel currents [45]. Furthermore, it has been revealed by mutagenesis that the presence of the residues K7 and P9 is essential for the κ-PVIIA–target interaction [62], as shown by NMR studies [40,49]. Recent studies provided evidence that the binding of κ-PVIIA to the *Shaker* K^+^ channel determines the behavior of the channel in both its conductive and not conductive states [63]. The fold adopted by κ-PVIIA is unrelated to any of the three other folds of toxins extracted from scorpions [64], sea anemones [65], and snakes [66], which block K_v_1 potassium channels. Nevertheless, κ-PVIIA also blocks human K_v_1.1 and K_v_1.4 channels, albeit with a lower potency compared to the *Shaker* K^+^ channel [47]. All these peptides, however, share a functionally significant dyad motif consisting of a crucial lysine that occludes the potassium channel pore and a hydrophobic, usually aromatic, residue, which plays a key role in the interaction with the target K^+^ channel [62,64,67]. Sudarslal and coworkers isolated a novel 13-residue peptide, called Mo1659, from the venom of *Conus monile*, consisting of an unusual sequence, with a preponderance of aromatic residues, an absence of apolar aliphatic residues, and no disulfide bridges [48]. Electrophysiological studies revealed the effect of Mo1659 on measured currents in dorsal root ganglion neurons, suggesting that the peptide targets non-inactivating voltage-dependent potassium channels. 

#### 2.1.2. κ-M-Conotoxins RIIIK and RIIIJ

**Figure 7:**
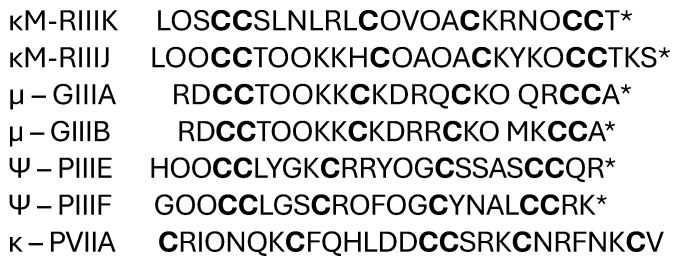
Sequence alignment of κM-conotoxins RIIIK and RIIIJ with other M-superfamily peptides and κ-conotoxin PVIIA. O represents 4-hydroxyproline and * an amidated C-terminal amino acid.

Venomous organisms produce a variety of structurally diverse peptide neurotoxins that target ion channels. The κ-M-Conotoxin RIIIK (κM-RIIIK) peptide is 24 amino acids long and is extracted from the cone snail *Conus radiatus.* It is structurally homologous to µ-conotoxin GIIIA, a well-known peptide that specifically blocks skeletal muscle voltage-gated Na^+^ channels (Figure 7) [68]. Ferber and coworkers showed that κ-M-RIIIK does not interact with Na^+^ channels but inhibits the *Shaker* K^+^ channels expressed in *Xenopus* oocytes in a state-dependent manner [69]. Functional and structural studies have revealed that κ-M-conotoxin RIIIK blocks voltage-activated K^+^ channels with a novel pharmacophore that does not comprise a dyad motif [70].

Furthermore, it was demonstrated that κM-RIIIK interacts with the channel’s pore area by employing *Shaker* channel mutants with single-residue substitutions. The introduction of a negative charge at residue 427 (K427D) significantly increased κM-RIIIK’s binding affinity [69]. κM-RIIIK was originally identified as a *Shaker* (*Drosophila*) and TSha1 (trout) K_v_1 orthologue channel blocker [69]. The *TSha1* K^+^ channel, which is the teleost homologue of *Shaker*, from CNS of *Oncorhychus mykiss*, is the highest-affinity target of кM-RIIIK that has been found thus far. The *TSha1* K^+^ channel was cloned and found to be the equivalent of mammalian K_v_1.2 channels [52]. In its outer vestibule, *TSha1* has a glutamate in place of K427 in the *Shaker* channel. According to its functional description, кM-RIIIK blocks the *TSha1* K^+^ channel in a state-dependent manner, with an IC_50_ of 20 nM in the closed state and 60 nM at 0 mV in the open state [52]. Remarkably, кM-RIIIK’s 24-amino acid sequence lacks an aromatic side chain but shows three positively charged residues. Four amino acids—L1, R10, K18, and R19—are necessary for K^+^ channel binding.

Later, κM-RIIIK became the first conotoxin identified to affect human K_v_1 channels, specifically inhibiting homomeric K_v_1.2 without any effects on Na_v_s or mammalian homologs K_v_1.1, K_v_1.3, K_v_1.4, K_v_1.5, and K_v_1.6, as measured by two electrode voltage clamp recording (TEVC) in *Xenopus oocytes* [71]. Heteromerization with K_v_1.2 α-subunits creates heterodimeric channels in K_v_1.1, K_v_1.5, and K_v_1.7 that are sensitive to low κM-RIIIK levels [51]. κM-RIIIK binds more strongly to closed (deactivated) K_v_1.2 channels than to open channels, suggesting state-dependent interactions between the peptide and K_v_1s [52].

M-conotoxin RIIIJ from the venom of the fish-hunting species *Conus radiatus* also represents a valuable inhibitor of voltage-gated potassium channels belonging to the K_v_1 subfamily. 

The effects of M-RIIIJ on different human K channel subtypes has been investigated using the Xenopus oocyte system. Specifically, κM-conotoxin RIIIJ (κM-RIIIJ) uncovered several important insights. κM-RIIIJ inhibits hetero-multimeric K_v_1.2 channels (three K_v_1.2 subunits plus one K_v_1.1 or K_v_1.6 subunit) with potency and a 100-fold selectivity over homo-tetrameric K_v_1.2 channels in a dose-dependent manner [53]. 

Although both peptides belong to the M superfamily, the sequences of κM-RIIIJ and κM-RIIIK within the first intercysteine loop are diverse [51]. Thus, chimeric peptides were synthesized, in which the residues in the first intercysteine loops of κM-RIIIJ and κM-RIIIK were switched. The resulting chimera RIIIKΔ9 shows that the L9K substitution in κM-RIIIK provides an increase in its affinity to a level closer to that of κM-RIIIJ, suggesting that K9 is a key determinant residue in these toxins for K_v_1.2 channel inhibition [53].

Using κM-RIIIJ in a fine and masterful experiment, Condeiro et al. [53] identified a specific subpopulation of mouse dorsal root ganglion neurons containing at least two functional K_v_1.2 channel complexes, a hetero-multimer targeted with high affinity by κM-RIIIJ, and a K_v_1.2 homo-tetramer, to which κM-RIIIJ binds less effectively. This example highlights the utility of conotoxins and other biologically active molecules of marine origin as pharmacological tools.

#### 2.1.3. Conkunitzin-S1

**Figure 8:**
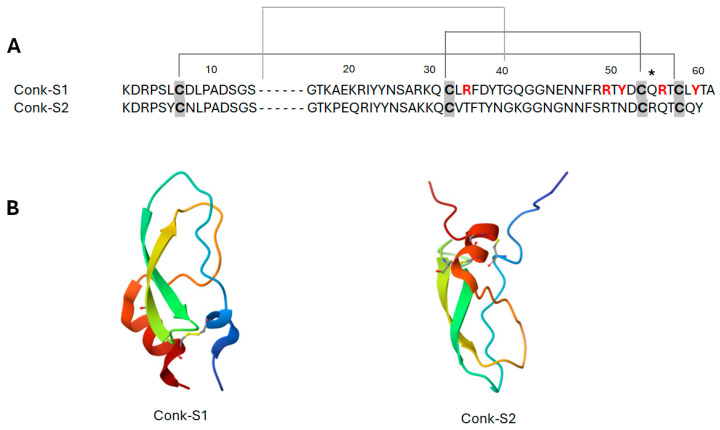
(**A**) Sequence alignment of chosen Kunitz-fold peptides (Conk-S1 and Conk-S2). Grey shading indicates conserved cysteine residues. Two preserved disulfide bridges are shown in solid lines. A dotted line indicates a third, non-conserved bridge. The secondary structure elements are illustrated at the bottom. A non-conserved arginine identified as a critical residue for channel block is denoted by an asterisk. The bioactive residues of both Conk-S1 and Conk-S2 from *Conus Striatus,* are highlighted in red. (**B**) Ribbon representations of Conk-S1 (PDB: 2CA7) and Conk-S2 (PDB: 2J6D). The yellow line represents disulfide bridges. The bioactive residues of Conk-S1 are highlighted in (**A**).

Conkunitzin-S1 (Conk-S1) is a 60-residue marine toxin identified from the venom of the cone snail *Cornus Striatus* that inhibits the K_v_1 channels in mammals and the *Shaker* channel [72]. Conk-S1 shares a sequence of homology with Kunitz-type proteins, but it contains only four cysteine residues (Figure 8), resulting in only two out of the three highly conserved disulfide bridges, which are typically found in these peptides [72].

Conk-S1’s crystal structure is a Kunitz-type fold with non-covalent contacts, two disulfide bridges, an NH_2_-terminal 3–10 helix, 2-stranded β-sheet, and a COOH-terminal α-helix that stabilize each other [73]. Conk-S1 interactions with the vestibule of the K_v_ channel are suggested by the mutation K427D of the *Shaker* K^+^ channel, which improves Conk-S1’s blocking efficacy by more than 2000-fold [54].

Recent structural, functional, and computational studies have identified a novel mechanism for K^+^ channel inhibition by Conk-S1 [55]. Rather than directly impeding the ion conduction pathway, Conk-S1 appears to bind and to disturb the structural water network, stabilizing the K_v_ channel’s active state. This compresses permeability and blocks the passage of K^+^ ions [55]. A block strategy would be applicable to K_v_1.7-mediated current inhibition, as well as other homo- and hetero-tetrameric K_v_1 channels, as evidenced by the discovery that heteromerization with K_v_1.7 improves Conk-S1’s affinity for K_v_1.2-containing hetero-multimeric K_v_ channels [74]. 

Clinically used anti-diabetic therapeutics that block K_ATP_ channels in islets induce insulin release independent of the blood glucose level. Therefore, these drugs frequently precipitate dangerous hypoglycemic episodes in patients. In contrast, Conk-S1’s potent and selective blockade of K_v_1.7 in pancreatic islets potentiates insulin release in a glucose-dependent manner [55], suggesting that the inhibitors of K_v_1.7 channels may not precipitate hypoglycemic episodes. Conk-S1 is therefore an interesting chemotype to design novel glucose-dependent insulin secretagogues to treat diabetes mellitus.

#### 2.1.4. CPY or Tyrosine-Rich Conopeptides

**Figure 9:**
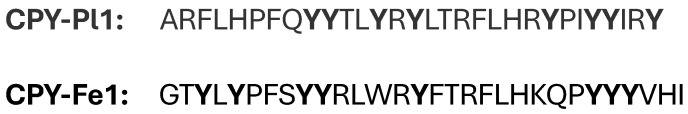
Molecular structures of CPY conopeptides. Tyrosine residues are evidenced in bold.

In 2008, Imperial and coworkers isolated and characterized two distinct peptides, named CPY-Pl1 and CPY-Fe1, from the venoms of sea snails *Conus planorbis* and *Conus ferrugineus*, respectively [56]. These peptides belong to a newly discovered family of conopeptides, named the conopeptide Y (CPY) family. Each peptide is 30 amino acids in length, lacks disulfide bridges, and is unusually rich in tyrosine, an unprecedented feature among native gene products (Figure 9). 

The abundance of tyrosine in CPY peptides might enhance their biological activity and specificity. Tyrosine residues are crucial for receptor binding, enzyme activity, and signal transduction, indicating that the unique tyrosine-rich composition of CPY peptides is likely vital for their pharmacological effects. Moreover, tyrosine engages in various interactions, such as hydrogen bonding, aromatic interactions, and phosphorylation, potentially affecting the peptide’s shape and pharmacological activity. Finally, the high tyrosine content in CPY peptides might reflect evolutionary adaptation and diversification within the Conus venom peptides, thus providing insights into the evolutionary tactics of venomous marine snails in developing bioactive peptides. 

These peptides have demonstrated pharmacological activity on specific mammalian K_v_1 channel isoforms. CPY-Pl1 features low nanomolar potency versus the K_v_1.2 isoform and high nanomolar potency versus K_v_1.6. On the other hand, CPY-Fe1 proved to be a selective nanomolar inhibitor of the K_v_1.6 isoform [56].

NMR spectroscopy revealed that while the peptides were unstructured in aqueous solution, a helical region formed in a trifluoroethanol buffer in one of the peptides, CPY-Pl1. Additionally, clones obtained from the cDNA of both species encoded prepropeptide precursors that shared an unique signal sequence, suggesting that these peptides are encoded by a novel gene family [56]. These features underscore the uniqueness and significance of CPY peptides in the study of Conus venom peptides.

#### 2.1.5. Conotoxin Pl14a

**Figure 10:**
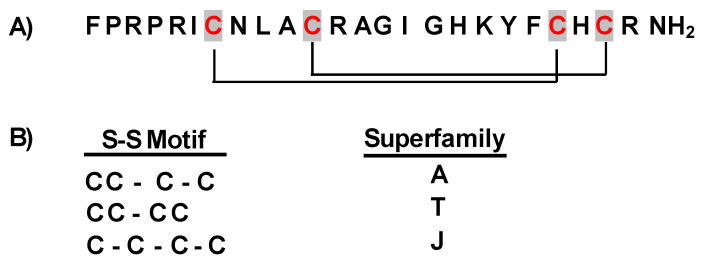
(**A**) Peptide sequence of pl14a showing the disulfide connectivity. (**B**) Disulfide motifs found in conotoxin superfamilies with four cysteines.

Assay-directed fractionation of venom from the vermivorous cone snail *Conus planorbis*, commonly found in the Indo-Pacific region, and classified in Clade IX, led to the isolation and characterization of a new conotoxin, named pl14a [57]. Pl14a has a C-C-C-C cysteine pattern and consists of 25 amino acid residues with variability in loop sizes and disulfide connectivity. It showed an amidated C-terminus, an extended N-terminal tail (six residues), and two disulfide bonds in a novel framework distinct from other conotoxins. The peptide was chemically synthesized, and its well-defined three-dimensional structure includes an alpha-helix and two 3–10 helices (Figure 10) [57].

Considering this feature, pl11a represents a new prepropeptide precursor of conotoxins, termed the J-conotoxin superfamily. All J-superfamily peptides belong to a distinct branch, separate from other conotoxins that share the same cysteine framework but have a different three-dimensional structure. These peptides share two structural features that are believed to influence their activity on K_v_1 channels. The first feature is an amino acid dyad, consisting of a positively charged residue (typically lysine), and a hydrophobic amino acid (usually aromatic), which protrudes from a relatively flat surface formed by the other residues of the peptide. The distance between the lysine α-carbon and the center of the aromatic ring is approximately 6–7 Å, with the lysine residue blocking the K^+^ channel pore. In some K_v_1 channel inhibitors lacking the functional dyad, a ring of basic residues on one surface of the molecule has been shown to facilitate binding to the outer vestibule of the channel. Pl14a contains both a potential dyad and a ring of basic residues, either of which may be crucial for its interaction with the K_v_1.6 channel. The discovery that pl14a affects both the K_v_1.6 channel and certain nAChR subtypes marks the first time a Conus peptide has been found to inhibit both voltage-gated and ligand-gated ion channels [57].

#### 2.1.6. Conotoxin sr11a

A new peptide blocker for K_v_1 potassium channels, known as conotoxin sr11a, was discovered in the vermivorous species *Conus spurius*. A lot of salient features of conotoxin sr11a have been discovered, and it shows multiple distinctions from other conopeptides that target K_v_ channels [58].

Peptide sr11a is a member of the I-conotoxin superfamily, from a vermivorous animal found in the Caribbean region of the West Atlantic, which is distinguished by its four disulfide bridges. It consists of 32 amino acid residues and has a pro-amidated C-terminus and two gamma-carboxy glutamates. It shares the characteristic pattern of the distantly related I-superfamily members [59]. In the structure of peptide sr11a, there is a lack of lysine residues, making it devoid of the functional dyad that is usually present in the peptides acting as K^+^ channels blockers. According to molecular modeling, the arginine residues R17 and R29 are components of the pharmacophore [58]. The Sr11a peptide’s impact on macroscopic K^+^ currents is assessed by applying fixed peptide concentrations and monitoring voltage–clamp currents [58]. Given peptide sr11a’s selective inhibition, it could be considered a potential drug candidate for modulating potassium currents and, for instance, for the treatment of conditions associated with potassium channel dysfunction. Comparing conotoxin sr11a to other conopeptides that target K_v_ channels, it shows a particular selectivity over K_v_1 isoforms, offering important insights into possible modes of action and therapeutic uses [75].

#### 2.1.7. Other Conotoxins Modulating K_v_s

**Figure 11:**
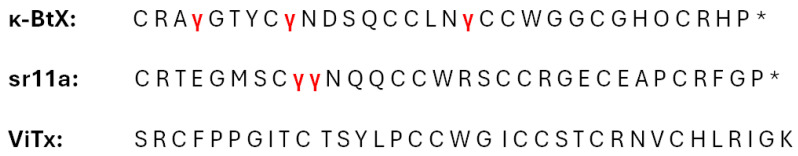
Comparison of peptide sr11a with other I-conotoxins from vermivorous species. γ, gamma-carboxy-glutamate; O, hydroxyPro; S, glycosylated Ser; *, amidated C-terminus [75].

The ĸ-BtX peptide, included in the I-superfamily, was characterized from the venom of *Conus betulinus* (Figure 11) [39]. This peptide was shown to upmodulate the Ca^2+^ and voltage-activated BK currents (KCa1.1) measured from rat adrenal chromaffin cells and did not affect other voltage-gated channels. Another member of the I-superfamily, designated ViTx from *Conus virgo* (Figure 11), has been shown to inhibit the K_v_1.1 and K_v_1.3 subtypes, but not K_v_1.2 [49,50,76]. In particular, at 0.8 µM, ViTx showed similar inhibition of K_v_1.1 and K_v_1.3 (33% and 36%, respectively), but was devoid of any effect over K_v_7.2 [49]. In the A superfamily, the ĸA-conotoxin SIVA was identified from the venom of the fish-hunting cone snail *Conus striatus* [77]. Recordings from the frog cutaneous pectoris muscle and principal neurons from the frog sympathetic ganglion reveal that this peptide induces repetitive activity in these cells. Furthermore, the *Shaker* K^+^-channels expressed in *Xenopus* oocytes are blocked by micromolar concentrations of ĸA-SIVA. However, the molecular identity of the vertebrate K^+^ channel target that showed high-affinity for the ĸA-SIVA peptide has not yet been identified.

### 2.2. Sea Anemones

Sea anemones are predatory marine animals that belong to the phylum *Cnidaria*, one of the oldest venomous lineages in existence. Sea anemones store venom in specialized stinging organelles known as nematocysts, which have venom-filled capsules to serve as chemical weapons. Contact with prey causes an explosive eversion of the tubule, piercing the target organism and releasing venom for predation, defense, or competitive deterrence [78]. They produce venoms of exceptional molecular diversity that contain various classes of peptide toxins, which play a critical role not only in the immobilization of prey and defense against predators but also in targeting a diverse array of ion channels [21,78,79]. These venom components have traditionally been classified according to pharmacological activity and amino acid sequence [78].

Due to the large diversity of toxins produced from sea anemone and both their functional convergence and promiscuity, the classification and characterization of sea anemone toxins has been performed opportunistically, comparable to many other venomous lineages, with an emphasis on readily accessible toxins and taxa that may have therapeutic value. Despite decades of investigation, the majority of sea anemone venom’s composition is still unknown [80]. Nevertheless, sea anemone venoms have been shown to be complex and rich sources of proteins, non-proteinaceous compounds, enzymes, and other proteins without enzymatic activity. The main components found in sea anemone venom are traditionally grouped into four functional types: 

(1) Phospholipases A_2_, which degrade membrane phospholipids of neuronal and muscle cells and are responsible for nerve damage and muscle inflammation [81]; 

(2) Cytolysins, which cause cell lysis by acting on cell membranes [82]; 

(3) Peptide neurotoxins, which interact with receptors, voltage-gated and ligand-gated ion channels, thereby altering neural transmission [21,83];

(4) Non-proteinaceous compounds (e.g., purines, biogenic amines), which are believed to induce pain during envenomation [84].

The K_v_ channels targeting sea anemone toxins all fall into the third group, peptide neurotoxins, which interfere with the transmission of nerve impulses by modifying the function of ion channels in nerve or muscle cells [85]. Nowadays, nine unique cysteine-rich peptide structural folds have been identified: β-defensin-like, boundless β-hairpin (BBH), epidermal growth factor-like (EGF-like), inhibitor cystine-knot (ICK), Kunitz-domain, proline-hinged asymmetric β-hairpin (PHAB), and small cysteine-rich peptides (SCRiPs) and ShK.

Recently, five peptide toxins, which structurally constitute a new family but target different ion channels, have been isolated: BDS-I and -II (K_v_3 potassium channel toxins) from *Anemonia sulcata*, APETx1 (human *ether-a-go-go*-related gene potassium channel toxin) and APETx1/2 (acid-sensing sodium channel toxin) from *Anthopleura elegantissima*. In addition, the following structurally novel peptide toxins have also emerged in sea anemones: gigantoxin I (epidermal growth factor-like toxin) from *Stichodactyla gigantea* and acrorhagins I and II from the acrorhagi (specialized aggressive organs) of *Actinia equina*. 

Another promising compound is ShK, originally isolated from the sea anemone *Stichodactyla helianthus*, which has been extensively studied for its potent inhibition of K_v_1.3 channels, predominantly expressed in T lymphocytes. ShK toxin inhibition of K_v_1.3 channels has implications for autoimmune disease, where dysregulated T cell activation plays a central role [86]. Table 2 summarizes the main sea anemones peptides acting on the different K^+^-channel targets and their inhibitory activities.

#### 2.2.1. β-Defensin-like Peptides

β-defensins are ubiquitous antimicrobial peptides that are secreted as part of the innate immune system and are used by anemones as neurotoxins. A ẞ-defensin fold is characterized by a short helix or turn, followed by a small twisted antiparallel beta-sheet [101]. Their high degree of specificity and selectivity has allowed them to modify the activity of voltage- and ligand-gated ion channels; hence, these toxins may provide valuable lead molecules for the development of new pharmaceuticals to block ligand-gated and voltage-gated ion channels [101].

**Figure 12:**
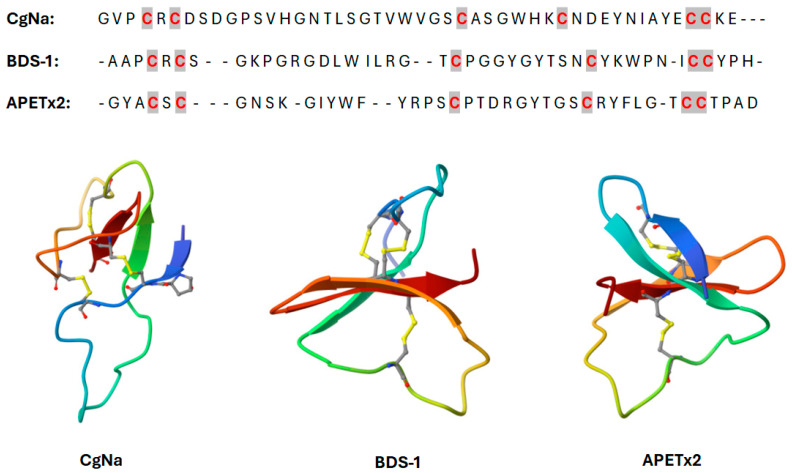
The sea anemone mature peptide sequences resemble β-defensin. The conserved cysteine residues are marked with red writing on a grey background. Tentacles, column, mesenterial filaments, and combinations are represented by letters T, C, F, and M, which are emphasized in blue, orange, green, and yellow, respectively. Homology modeling predicts various mature peptides from sea anemones using CgNa (PDB 2H9X), BDS-I (PDB 1BDS), and APETx2 (PDB 2MUB).

In a recent study, Qiqi Guo and coworkers have found 11 homologous sequences to sea anemone toxin’s β-defensin-like peptides with the cysteine pattern CXC-C-C-CC [102]. The fundamental β-defensin structure is based on disulfide bridges between cysteine residues C1-C5, C2-C4, and C3-C6. According to sequence similarity analysis, the sequence identities of HC-71 and Rc I (GenBank No. P0C5G5.1), HC-66/70, and Am II (GenBank No. P69930.1) were 97.87%, 97.83%, and 95.65%, respectively. Using CgNa (PDB 2H9X), BDS I (PDB 1BDS), and APETx2 (PDB 2MUB) as templates, homology modeling prediction revealed that HC-71 and Rc I, HC-64/68 and BDS I, and HC-65/69 and APETx2 had similar 3D structures (Figure 12). As a result, these peptide toxins may inhibit multiple ion channels, including Na_v_, K_v_, and ASIC.

**Figure 13:**
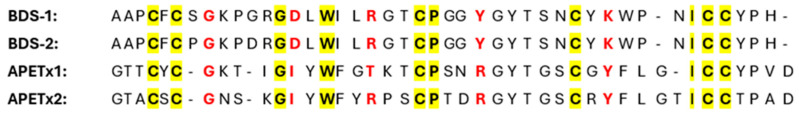
Amino acid sequences of a new family of sea anemone peptide toxins. Charged residues are bolded in red. The sequence identities are highlighted in yellow.

The K_V_ type 3 sea anemone toxins are composed of 42–43 residue β-defensins and include, among others, Blood Depressing Substance I (Δк-actitoxin-Avd4a, BDS-I) and BDS-II from *Anemonia sulcata* [87], as well as APETx1/2 (к-actitoxin-Ael2a, π-actitoxin-Ael2b) from *Anthopleura elegantissima* (Figure 13) [89]. 

BDS toxins with a total length of 43 amino acids were first described as antihypertensive and antiviral peptides. BDS toxins include BDS-I and BDS-II, which show the 93% sequence identity, with only two differences in the amino acidic sequence (positions 7 and 11). They have been found to be blockers of the K_v_3.1, K_v_3.2, and K_v_3.4 potassium channels at nanomolar concentrations, while at high concentrations they weakly inhibit K_V_1.1–5, K_V_2.1–2, K_V_4.1 and K_V_4.3 channels [87,103,104,105]. Indeed, the differences between the two toxins affect only positions 7 and 11 (Figure 13). Intriguingly, it has been demonstrated that BDS-I is a selective blocker of the fast-inactivating K_v_3.4 subfamily, showing no discernible blocking activity against the K_v_1, K_v_2, or K_v_4 families, or against any other members of the K_v_3 family [88,106].

This is why BDS toxins are increasingly being utilized to clarify the role of the K_v_3.4 channel, which is implicated in two major disorders of the central nervous system: Parkinson’s disease and Alzheimer’s disease [107,108]. 

APETx1 contains 42 amino acid residues, and it is a potent and selective blocker (IC_50_ = 34 nM) of the human ether-a-go-go-related K^+^ channel (hERG), encoded by the ether-a-go-go-related gene (hERG) [89]. Given that hereditary hERG gene mutations are known to cause the long QT syndrome, which involves ventricular fibrillation and episodic ventricular arrhythmias that can eventually lead to syncope and sudden death, hERG channels are essential in cardiac cells [109,110]. Only hERG1 of the three hERG isoforms (hERG1, hERG2, and hERG3) was previously known to be sensitive to APETx1 [111]. However, a recent study found that hERG2 is insensitive to APETx1, whilst hERG1 and hERG3 respond equally to it [90]. As evidenced by the cysteine-scanning analysis of hERG, the binding site of APETx1 is localized in the S3–S4 region of the VSD domain [112]. 

On the other hand, APETx2 (π-actitoxin-Ael2b) is a peptide toxin with 42 amino acid residues, having antibacterial and neurotoxin activity. It belongs to the disulfide-rich all-β structural family, with a fold typical of the β-defensin family [113]. It is functionally quite unique because it does not inhibit potassium channels but rather acid-sensing ion channels (ASICs Hþ-gated sodium channels), which are mainly expressed in central and peripheric nervous systems where they function as pain sensation modulators [114]. 

#### 2.2.2. Inhibitor Cystine-Knot (ICK) Motif

**Figure 14:**
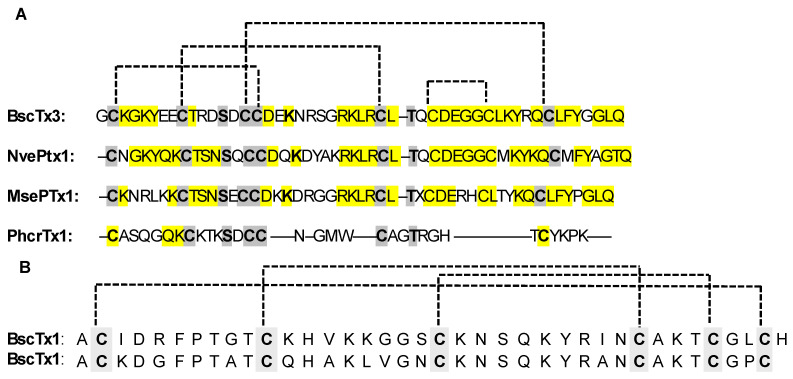
(**A**) Alignment of sea anemone toxins, which most likely have an ICK fold. Predicted disulfide connectivities are illustrated with dotted lines. Amino acid identities are highlighted in yellow and similarities in bold. The toxin sequences given are BcsTx3 (κ-actitoxin-Bcs4a; UniProt C0HJC4), NvePTx1 (U-EWTX-NvePTx1; UniProt A7RMN1), MsePTx1 (U-metritoxin-Msn2a; UniProt P0DMD7), and PhcrTx1 (π-phymatoxin-Pcf1a; UniProt C0HJB1) [78]. (**B**) Structures of BscTx1 and BscTx2 type-1 toxins. Disulfide bridges are illustrated by dotted lines.

Venomous animals’ anemone toxins contain the ubiquitous inhibitor cystine knot (ICK) motif, which is one of the most frequently recruited peptide folds [115]. The ICK family consists of structural peptides that act as anti-pathogen defenses by binding to ion channels. ICK is widely distributed across many species, and ICK toxins are frequently present in animal venom, where they aid in defense and predation. Although the 3D structure of putative sea anemone ICK toxins needs to be validated, two distinct types of sea anemone toxins have cysteine patterns resembling the ICK fold shown in Figure 14: ASIC toxin PhcrTx1 (π-phymatoxin-Pcf1a) and the BcsTx3 toxin. Although the disulfide framework and 3D structure have yet to be identified, the distribution of cysteines follows the “classic” ICK signature CXnCXnCCXnCXnC, where “X” is any amino acid and “n” represents a variable number of amino acid residues [115]. 

PhcrTx1 was the first peptide characterized from the venom of *Phymanthus crucifer* showing a high affinity for the ASIC channels [116]. It is a 32-residue peptide with three disulfide bonds that reversibly inhibits ASIC currents in rat dorsal root ganglia neurons with an IC_50_ of 100 nM [92]. BcsTx3, a K_v_ channel blocker originating from *Bunodosoma caissarum*, is a typical ICK representative [116]. Usually, toxins are classified into four different types (types 1–4), according to their differences in primary structures, disulfide bridge patterns, and pharmacological activity. BcsTx3 has been defined as the very first representative of type 5 toxins. The BcsTx3 disulfide bridge pattern has not been determined yet; however, its primary structure and the traditional ICK framework suggest four disulfide bridges, if compared to the three disulfide bridges evidenced for ICK analogues from the venoms of *Nematostella vectensis* (NvePTx1) and *Metridium senile* (MsePTx1) (Figure 14) [92]. Based on the cysteine pattern and sequence similarities, they are likely new members of the K_v_ type 5 toxins family, but their presence in venom is still unproven (Figure 14).These structural features probably account for the pharmacological activity of the peptide toxin which, besides being able to inhibit K_v_1.3 and K_v_1.6, is much more selective for K_v_1.2 and, in particular, *Shaker* channels (Table 2). This activity profile is probably the reason why, in *Bunodosoma caissarum* venom, BcsTx3 coexists with BcsTx1 and BcsTx2, (Figure 14), exerting complementary K_v_ channels selectivity. BcsTx1 and BcsTx2 are two type-one toxins, characterized by a key basic residue (K) associated with a 6.6 ± 1 Å distant key aromatic residue (Y) [91]. The side chain of the K residue is responsible for the interaction with the ion channel pore, while an aromatic residue interacts through both electrostatic forces and hydrogen bonding with a cluster of aromatic residues in the P-loop region [92]. While showing inhibitory activities over K_v_1 and *Shaker* channels (Table 2), BcsTx1 is particularly interesting due to its selectivity for the K_v_1.2 channel.

#### 2.2.3. Kunitz-Type Peptides

**Figure 15:**
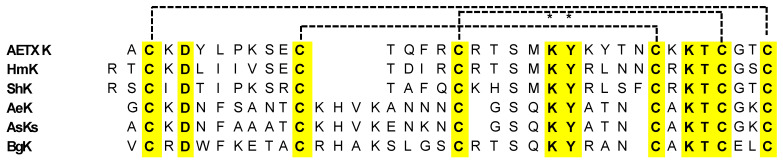
Alignment of the amino acid sequence of AETXk with those of the known type 1 potassium channel toxins from sea anemones: HmK, ShK, AeK, AsKS, and BgK. The residues identical with AETXk are highlighted. Disulfide bridges are depicted by dotted lines. Asterisks represent the amino acid dyad that is crucial for the binding to potassium channels.

Sea anemones are well known to produce different toxins, mostly belonging to the Kunitz-type family, mainly acting as serine protease inhibitors, but also showing inhibition of the currents evoked by various potassium ion channels, particularly in excitable cells [93,117]. The toxins can be grouped into four structural classes: type 1, with 35–37 amino acid residues and three disulfide bridges; type 2, with 58–60 residues and three disulfide bridges; type 3, with 41–42 residues and three disulfide bridges; and type 4, with 28 residues and two disulfide bridges. The interest in the Kunitz-type peptides family is determined by the broad representation of these peptides in living organisms, as well as by their structural diversity and multitargeting. They are ubiquitous in marine organism venom, exerting multiple biological activities. Some of these peptides differ by a single amino acid substitution, and in addition to their protease inhibitory activity, they can also block voltage-gated potassium (K_v_), calcium (Ca_v_), and acid-sensing ion (ASIC) channels. Examples from the type I Kunitz-domain sea anemones toxin targeting potassium channels are ShK from *Stichodactyla helianthus*, BgK from *Bunodosoma granulifera*, HmK from *Heteractis magnifica*, AETXk from *Anemonia erythraea*, and AeK from *Actinia Aequina* (Figure 15).

These peptides are characterized by the six conserved cysteines that form three disulfide bonds (C1–C6, C2–C4, C3–C5), a highly conserved aspartic acid residue (D4/5), the KY dyad, and the KTC motif close to the N-terminus (Figure 15) [118]. 

**Figure 16:**
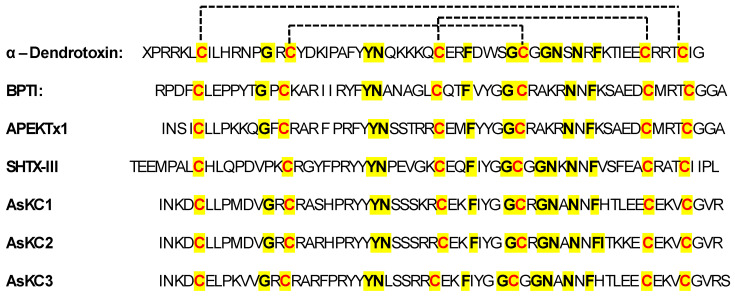
Amino acid sequences of Kuntitz-type peptides APEKTx1, SHTX-III, and AsKC1–3. Shaded areas indicate spots that are highly conserved. α-dendrotoxin (a K^+^ channel-blocking toxin from the green mamba *Dendroaspis angusticeps*) and BPTI (bovine pancreatic trypsin inhibitor, the first-described Kunitz protein) are depicted as reference compounds. The three disulfide bridges are represented by a dotted line, and the cysteine residues involved are highlighted in red [119].

On the other hand, the type-2 Kunitz peptides consist of about 60 amino acid residues. In addition, for these peptides, the bonding between the α and β subunits folding is stabilized by three conserved disulfide bonds (C1–C6, C2–C4, C3–C5, Figure 16) [117]. The peptides show highly conserved residues, particularly glycine, asparagine, and phenylalanine residues, as shown in the sequences depicted in Figure 16.

##### ShK Peptide

ShK, a 35-reside peptide isolated from the Caribbean Sea anemone Stichodactyla helianthus, blocks K+ channels (K_v_1.1, K_v_1.3, K_v_1.6, K_v_3.2, KCa3.1) and exhibits low picomolar to nanomolar affinity [98]. Since the blockade of Kv1.3, a channel expressed in T lymphocytes, suppresses T cell activation, considerable effort has been expended to develop K_v_1.3-selective ShK analogues for use as immunomodulators. Using a synthetic approach, ShK-analogues (ShK-186/dalazatide, ShK-EWSS, ShK-K-amide, ShK-192, and ShK-K18A) with picomolar affinity and exquisite selectivity for K_v_1.3 were developed [120,121,122,123,124,125,126,127,128]. ShK physically obstructs ion permeation by binding to the outer pore of Kv1.3 channels. Cryo-EM studies with ShK-186/dalazatide and ShK-Fab revealed that ShK binds to K_v_1.3’s outer vestibule in the C-type inactivated conformation, narrows the outer pore by flipping Y447 towards the interior of the selectivity filter, and stabilizes the closed conformation through a network of inter-subunit hydrogen bonds [129,130]. Molecular dynamics simulation studies based on the cryo-EM structures have highlighted the role of conformational dynamics in the interaction of ShK with KV1.3’s outer vestibule [131].

Activated auto-reactive effector memory T (TEM)-cells are implicated in the pathogenesis of many autoimmune disorders. K_v_1.3 is up-regulated in activated TEM cells and plays a critical role in regulating membrane potential and calcium signaling in this subset of T cells. Blockade of K_v_1.3 in TEM cells has been reported to suppress cytokine production, migration, and proliferation in vitro and ameliorate disease in animal models of multiple sclerosis, rheumatoid arthritis, and atopic dermatitis [130]. ShK-186/dalazatide has advanced to clinical trials. In animal models of autoimmunity, it exhibited a long-lasting therapeutic effect due to its high selectivity, tight binding and slow release from K_v_1.3 channels on T cells [120,121]. In a phase 1 clinical trial, it ameliorated plaque psoriasis in patients [123]. Other K_v_1.3 inhibitors are in various stages of pre-clinical and clinical development.

##### BgK Peptide

BgK is a potassium channel toxin composed of 37 amino acids containing three disulfide bridges (C2–C37, C11–C30, and C20–C34), which are essential to the toxin’s structure and activity, especially concerning the inhibition of K_v_l channels [131].

The disulfide bridges are arranged in a precise way that guarantees the toxin’s optimal folding into a compact and stable conformation, which is necessary for its biological function of BgK [131]. 

BgK binds to and blocks the K_v_1.1, K_v_1.2, K_v_1.3, and K_v_1.6 channels with similar potency, but does not affect the K_v_1.4 and K_v_1.5 channels [97]. The selectivity of BgK for K_v_1 can be modified by F6A replacement, which reduces its affinity for homomultimeric K_v_1.2 and K_v_1.3 without affecting its affinity for homomultimeric K_v_1.1 [97]. This selectivity shift improves neurological outcomes, including brain damage and energy metabolism, while not affecting T-cell activation. The findings provide new suggestions for the use of BgK-F6A as a scaffold for drug design in neuro-inflammatory diseases, including multiple sclerosis and stroke [132]. The second key factor for BgK binding involves hydrophobic interactions of its functional dyad (K25 and Y26) with the K_v_1.1 Y379 residue, which protrudes into the channel vestibule. Interestingly, BgK binds more tightly to K_v_1.1, K_v_1.2, and K_v_1.5, where residue 379 is either a tyrosine or a valine, than to K_v_1.3, where this residue is replaced by histidine, which is less hydrophobic. In fact, BgK has a strongly reduced affinity for Y379H K_v_1.1 mutants and a comparable increased affinity for H379Ymutants [133]. 

##### HmK Peptide 

HmK has been isolated from the tropical sea anemone *Heteractis magnifica* [134]. HmK has an identical molecular weight (4055 amu) to ShK, exhibits 60% homology with ShK, and shares approximately 40% identity with BgK. Similar to its homologues, HmK shows three disulfide bridges (C3-C35, C12-C28, and C17-C32), resulting in a similar secondary structure, characterized by three short helices [134]. It also possesses the functional dyad K22-Y23. Nevertheless, HmK is almost 300-fold less potent than ShK (Table 2). Molecular docking studies revealed that HmK is probably penalized in the interaction with K_v_1.3 by its greater rigidity, compared to ShK, limiting the number of accessory interactions occurring at the binding site, and thus, penalizing the binding energy of HmK/K_v_1.3 complexes [135].

##### AETXk Peptide 

AETXk is another relevant member of the type 1 Kunitz-type potassium channel toxins from sea anemones, exhibiting high sequence identities with HmK and ShK. It has the typical cysteine patterns of its analogues, as well as the functional dyad (Figure 15) [99]. 

Though far less powerful than the previously discussed peptides (Table 2), AETX K also prevents the binding of ^125^I-α-dendrotoxin to rat synaptosomal membranes. On the other hand, AETXk acts as a *Shaker* channel inhibitor, similar to HmK, though with lower affinity. Additional investigation into the molecular processes behind these differences may shed light on the many ways in which sea anemone poisons affect potassium channels [99].

##### AeK Peptide 

The aqueous extract of Actinia equina, a relatively small species found in Japan’s coastal waters, has been recently reported to exhibit potassium channel toxicity [100]. The characterization of the extract identified a potassium channel toxin, named AeK, that exhibits the ability in inhibiting the binding of ^125^I-α-dendrotoxin to rat synaptosomal membranes in a dose-dependent manner. 

The sequence homology of AeK with AsKS is as high as 86%, while the homologies with BgK and ShK are moderate (53%with BgK and 36% with ShK). Importantly, AeK has six cysteine residues at the same positions as the other type-1 Kunitz-like toxins. This strongly suggests that the three disulfide bonds of AeK, though not yet assigned, are located between C2–36, C11–29, and C20–33, as previously reported for BgK and ShK. Furthermore, it is interesting to note that the two amino acid residues (K22 and Y23), which have been assumed to be the most essential residues for the binding of ShK with potassium channels, are conserved in AeK, as well as in BgK. Selectivity profiles of AeK over the different K_v_1 isoforms are still lacking, and this is a matter worth investigating in the near future [100].

##### Type-2 Kunitz Peptides 

The bovine pancreatic trypsin inhibitor (BTPI)-Kunitz-type protein ShPI-1 is the major protease inhibitor from the sea anemone *Stichodactyla helianthus,* containing 55 amino acids cross-linked by three disulfide bridges. Because of its anti-parasitic properties, this protein is employed in biotechnology and may have applications in biomedicine [136]. ShPI-1 acts as a proteases inhibitor, targeting serine proteases, cysteine proteases, and aspartic proteases, although it shares structural homology with both the Kunitz-type protease inhibitor BPTI and the snake dendrotoxins (DTXs), which are powerful blockers of voltage-gated potassium channels (K_V_) [137]. It has been demonstrated that five related proteins from sea anemones have two functions because they block both K_v_ channels and serine proteases. The first bifunctional BPTI-Kunitz-type proteins to be identified from sea anemones are called kalicludines (AsKC1–AsKC3), from *Anemonia sulcata* [93].

Similar actions were later reported for APEKTx1, from *Anthopleura elegantissima*, and SHTX-III, from *Stichodactyla haddoni* [94,138]. The selectivity of these toxins for the K_v_ channel subtypes is generally unknown. A comprehensive examination of several ion channels, initially reported for APEKTx1, demonstrated strong selectivity and efficacy against K_v_1.1, with IC_50_ values in the lower nanomolar range [94]. Venoms have long been regarded as a viable source of therapeutic possibilities due to their great molecular specificity and potency. These compounds are also useful for developing new drugs and for researching the composition and the performance of potassium channels.

Type-2 Kunitz-type peptides, known as Kalicludines (AsKC1–3), have also been isolated and characterized in *A. sulcata* [139]. These peptides are a class of sea anemone toxins that possess dual activities: blocking the voltage-sensitive K_v_1 channels and inhibiting trypsin. These unique properties are attributed to their structural homologies with serine protease inhibitors of the Kunitz type and dendrotoxins. Kalicludines are 57–60 amino acid peptides with 40–41% homologies with the Kunitz inhibitor and 38–42% homologies with DTX I, a potent blocker of the K_v_1.2 channel [139].

The inhibitory effects of Kalicludines on voltage-sensitive K_v_1 channels are demonstrated by their ability to inhibit ^125^I-DTX binding to K_v_ channel proteins and block the K_v_1.2 channel currents evoked in Xenopus oocytes [93]. The structural specificity of this class of compounds for K_v_1.2 has not been fully explained so far, and would be a pivotal starting point for the development of selective K_v_1.2 channel inhibitors

#### 2.2.4. Proline-Hinged Asymmetric Β-Hairpin (PHAB)

**Figure 17:**
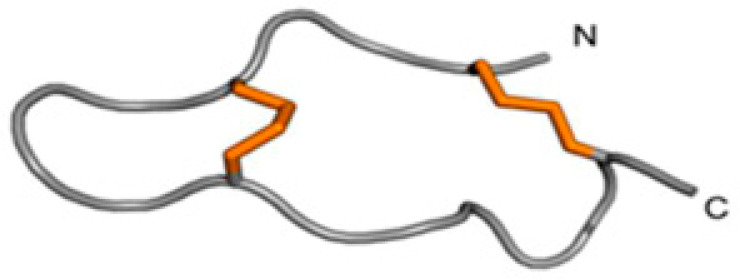
Disulfide bonds in Ate1a (PDB 6AZA) are depicted as orange tubes, with N- and C-termini labeled [55].

**Figure 18:**
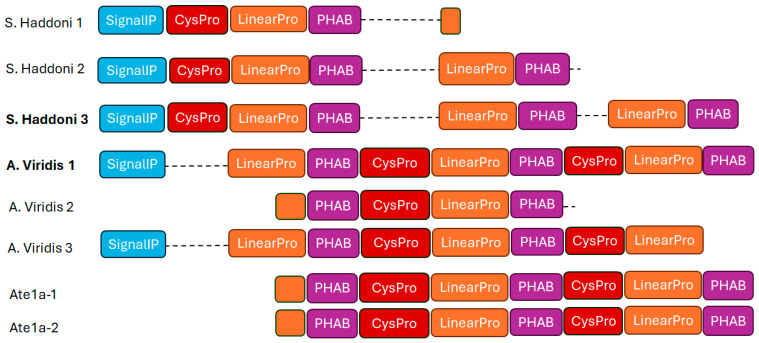
Domain structure of Ate1a and Ate1a-like contigs. Prepropeptides consist of a signal peptide (SignalP), one or two cysteine-containing propeptide domains (CysProP), and three cysteine-free propeptide domains (LinearProP), each preceding an Ate1a-like PHAB domain [95].

Potassium channel-blocking sea anemone toxins, or PHAB toxins, are a class of toxins present in marine animals, especially sea anemones. It is well known that these PHAB toxins can interact with and block several kinds of potassium channels in a variety of creatures, including humans. Potassium channels are essential for controlling how electrical activity is generated by all types of cells, including muscles and neurons. Depending on the potassium channels targeted and the organism exposed, PHAB toxins can cause a variety of effects by selectively blocking these channels and disrupting normal cellular activity. Potassium channels can be affected by PHAB toxins in a variety of manners [78]. They cause paralysis or other neurological consequences by obstructing the neurons’ normal capacity to conduct electrical messages. They could also interfere with muscular function and cause symptoms like weakness or spasms. Their potential medical use has compelled researchers to investigate PHAB toxins for the development of new pharmaceutical treatments to address difficult-to-treat diseases, including cardiac arrhythmias and neurological conditions. Furthermore, research on PHAB toxins can advance the knowledge of the variety of bioactive substances present in marine animals and their possible functions in marine ecosystems [95]. Two unusually short toxins, composed of only 17 amino acid residues, were recently described from the venoms of *Actinia tenebrosa* and *A. bermudensis*. These toxins are *named* Ate1a (κ-Actitoxin-Ate1a) and AbeTx1 (κ-Actitoxin-Abe1a), respectively [95,96]. The structural characterization of the first of these, Ate1a, revealed a previously undescribed fold comprising a β-hairpin-like topology stabilized by two disulfide bonds (Figure 17 and Figure 18) [96]. 

However, the hairpin’s two sides are uneven in length, with the longer side possessing a small three-residue proline hinge that most likely inhibits the formation of any secondary structure. The proline-hinged asymmetric β-hairpin (PHAB) fold differs from other hairpin-like peptide folds, including β-hairpin antimicrobial peptides (AMPs) or the cystine-stabilized α/α (CSαα) fold. Both Ate1a and AbeTx1 inhibit specific subtypes of K_V_ channels (K_V_1.1, K_V_ 1.2, and K_V_ 1.6). AbeTx1 has been found to interact with the outer vestibule of these channels through pivotal interactions exerted by residues K3, K7, and R11 [96]. Although PHAB toxins have only been found in the venoms of *A. bermudensis* and *A. tenebrosa*, they have also been found in the transcriptomes of *Stichodactyla haddoni* and *Anemonia viridis*, implying that they are prevalent throughout *Actinioidea*. All PHAB toxins discovered so far are encoded as numerous repeats of identical mature peptide sections on single transcripts. Interestingly, the sequence identities of these mature peptide domains appear to be conserved by intra-gene coordinated evolution, a highly rare phenomenon that has not been observed in any other toxin gene. A combination of toxicity studies and mass spectrometry imaging (MSI) indicated that Ate1a is almost exclusively localized in the tentacles, lacks antimicrobial activity, and most likely serves a predatory function by inhibiting prey K_v_ channels. The intra-gene concerted evolution of these toxins is thus most likely a strategy for maintaining effective expression levels of an ecologically significant toxin [92,95].

#### 2.2.5. kP-Crassipeptides

**Figure 19:**
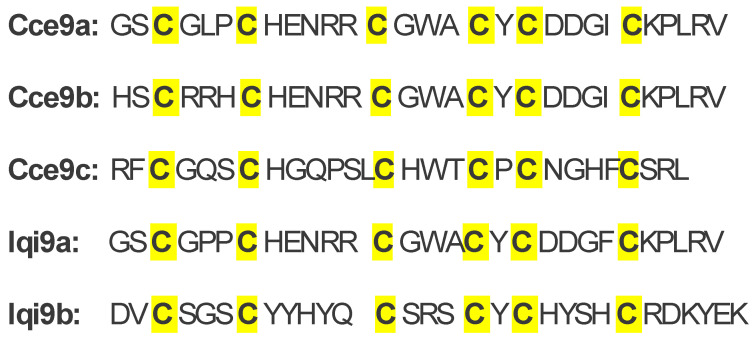
Primary sequence of the kP-crassipeptides. Cysteine residues are highlighted and aligned.

One of the most biodiverse invertebrate lineages in the marine environment encompasses the venomous marine snails in the superfamily Conoidea, a group that by current estimates comprises more than 10,000 species. These conoideans (also known as crassispirines or crassispirine snails) have generally been understudied, and their toxicology is largely unknown [140]. The first family of venom peptides from the crassispirines, the kP-crassipeptides, known as cce9a, cce9b, cc9c, iqi9a, and iqi9a, are functionally analogous to the various families of κ-conotoxin (κ-, κM-, and κJ-conotoxin families) precursors [140]. Initially, only cce9a was discovered from crude venom of Crassispira cerithina, back in 1838, and was only synthesized and characterized in 2011 [141]. Lately, the remaining peptides (Figure 19) have also been identified, and three of them (cce9a, cce9b and iqi9a ) were subjected to chemical synthesis and characterization, along with preliminary pharmacological evaluation [140].

Crassipeptides exhibit a conserved arrangement of cysteine residues in their primary sequence, which is similar to the cysteine framework found in the P-conotoxin family. Moreover, the precursor sequences of crassipeptides include a signal sequence, an intervening propeptide sequence, and a mature peptide sequence at the C-terminus. This arrangement is also a common feature in conotoxin precursors, which have approximately 25–30 amino acids and three disulfide bonds. This evidence suggests that crassipeptides are characterized by the typical three disulfide bond pattern evidenced for conopeptides [140]. Using a constellation pharmacology approach, the synthesized peptides cce9a, cce9b, and iqi9a were found to induce excitatory effects in a subset of cultured dorsal root ganglion neurons, suggesting a potential role in modulating neuronal activity. However, when tested using electrophysiology, only cc9b showed direct inhibition of the human K_v_1.1 channel (IC_50_ = 2.9 μM) and of the Drosophila *Shaker* K channel (IC_50_ = 1.1 μM). Other hK_v_1 channels tested (hK_v_1.2, hK_v_1.3, hKv1.4, hK_v_1.5, hK_v_1.6 and hK_v_1.7) were unresponsive to cce9b at concentrations up to 1 μM.

Thus, the K-channel blocking activity of the crassispirine snails diverges from the other K-channel blocking peptides previously characterized from *Conus* venoms, despite the structural similarities evidenced. Thus, the characterization of the crassipeptides opens up a new paradigm for the discovery and characterization of novel pharmacologically active venom components.

## 3. Marine Sponges

In addition to showing several different biological activities [142,143,144], it has been demonstrated that molecules originating from sea sponges possess the ability to influence potassium channels [145].

Bioactive substances with a wide range of molecular structures, including steroids, peptides, alkaloids, and terpenoids, are abundant in marine sponges. These substances can interact with potassium channels in multiple manners:

(1) Blocking: Some compounds generated from sponges have the ability to block potassium channels, hence obstructing ion passage. Cell excitability and signaling may change as a result. 

(2) Modulating: by altering their sensitivity to voltage or other ions, certain substances can modify the activity of potassium channels. 

(3) Opening: potassium channels can be opened by certain chemicals, which increase ion flow and influence biological functions such as neuronal firing and muscular contraction. 

**Figure 20:**
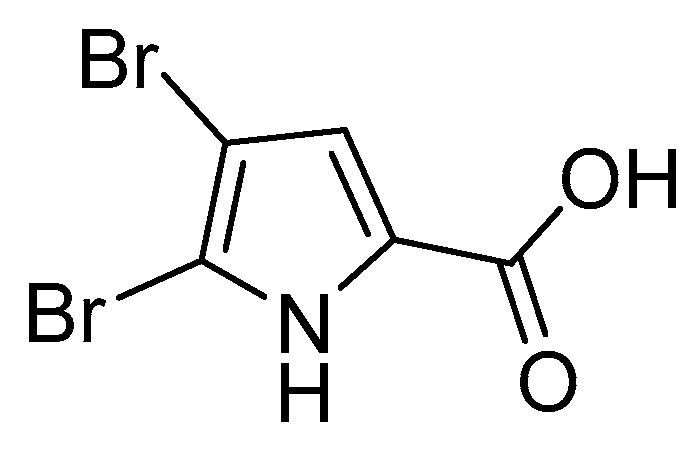
Structure of 4,5-dibromopyrrole-2-carboxylic acid.

Historically, natural compounds have been used as possible sources of lead compounds for medicinal research [146]. Between 1950 and 2019, almost 9500 novel compounds were identified from marine sponges, the majority of which displayed a broad spectrum of biological functions. Among the most prevalent sponges in tropical and subtropical regions worldwide, the genus *Agelas* of marine sponges (class *Demospongiae*, order *Agelasida*, family *Agelasidae*) are widely distributed in tropical and subtropical waters, becoming a distinctive and relatively unexplored source of natural products, with remarkable molecular diversity and a plethora of intriguing biological activity [147]. The mixed biogenetic origin of many of these metabolites is demonstrated by their complex structures, including alkaloids, glycosphingolipids, sterols, carotenoids, and so on. The genus *Agelas* of sponges is an attractive source for the discovery of fascinating natural chemicals acting on potassium channels. One prominent alkaloid is bromopyrrole alkaloid, 4,5-dibromopyrrole-2-carboxylic acid (Figure 20). 

### 3.1. Pyrrole Alkaloids

**Figure 21:**
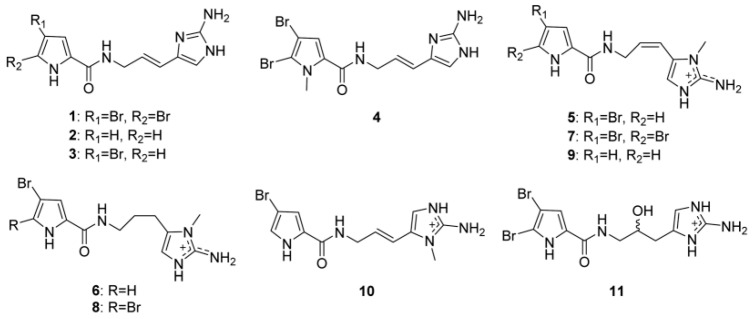
The chemical structures of pyrrole alkaloids **1**–**11** from Agelas sponges.

Pyrrole alkaloids are a fascinating class of a multitude of secondary metabolites produced by Agelas sponges. Because of their intriguing bioactivities and high chemo diversity, they have a long history of significance in the chemistry of marine natural products. In terms of structure, most of them have a bromo- or debromopyrrole-2-carboxamide core, which is joined to fused cyclic systems (fused cyclic pyrrole alkaloids) and a variety of linear side chains (linear pyrrole alkaloids). Moreover, a wide range of dimers (dimeric pyrrole alkaloids) can be produced by the dimerization of the monomers. It is crucial to acknowledge that these pyrrole alkaloids cover a wide range of chemical structures, yet their biosynthetic origins can be linked to a limited number of potential precursors. The extraordinary logic of nature dictates that only a few basic amino acids, such as proline and lysine, are required to make the simple pyrrole alkaloids (such as oroidin), which serve as building blocks for complex pyrrole alkaloids with a variety of molecular structures. Pyrrole-imidazole alkaloids (PIAs), which are composed of an aminoimidazole group coupled to an aliphatic tract by a bromo- or debromopyrrole-2-carboxamide core, account for the majority of linear pyrrole alkaloids. Produced only by marine sponges, these alkaloids have been considered valuable chemotaxonomic markers for axinellid sponges, which were formerly associated with the family Agelasidae, including the genera Agelas, Axinella, Acanthella, Hymeniacidon, Phakellia, and Pseudaxinyssa. Agelas sponges have also been observed to produce linear pyrrole alkaloids that do not have an aminoimidazole component in their molecules (Figure 21).

The most studied and diffused alkaloids from the *Agelas* sponges are clathrodin, hymenidin, and oroidin; these compounds, and their synthetic analogues, have been evaluated for their inhibitory effect on six isoforms of the K_v_1 subfamily of voltage-gated potassium channels, K_v_1.1-K_v_1.6 [145]. Because of their relatively simple structures and lead-like properties, clathrodin, hymenidin, and oroidin represent interesting starting compounds for medicinal chemistry optimization of their pharmacological properties.

In addition, clathrodin, hymenidin, oroidin, and their synthetic analogues were tested for their inhibitory activity on six different human K_v_1 channel subtypes expressed in *Chinese hamster* ovary cells (CHO cells), using automated patch clamp electrophysiology. All the compounds were evaluated against K_v_1.3 channels, and the most active were also tested on K_v_1.1, K_v_1.2, K_v_1.4, K_v_1.5, and K_v_1.6 channels. In addition to being evaluated using automated patch clamp electrophysiology, the compounds were also studied in a second, independent test system, under voltage clamp conditions, on K_v_1.1-K_v_1.6 and K_v_10.1 channels expressed in *Xenopus laevis* oocytes. Based on the inhibitory activities, the structure-activity relationship (SAR) of this new structural class of voltage-gated potassium channel modulators was studied [98]. 

Oroidin (Figure 21, compound **1**) was the first PIA to be isolated, and it was obtained from *A. oroides*, collected in the Bay of Naples in 1971 [148]. Three closely comparable counterparts have now been found: the monobrominated hymenidin (Figure 21, compound **3**), the N-methylated derivative sventrin (Figure 21, compound **4**) from Bahamas *A. sventres* [149], and the nonbrominated clathrodin (Figure 21, compound **2**) from Caribbean *A. clathrodes* collected from Desecheo Island [98]. The most basic PIAs, oroidin (compound **1**), clathrodin (compound **2**), and hymenidin (compound **3**), differ only in their bromine concentration. These three oligomeric building blocks are essential for the biosynthesis of several chemically complicated PIAs, with remarkable molecular diversity.

While compound **2** (clathrodin) has no effect on K_v_1.1–1.6 channels, compound **1** (oroidin) and compound **2** (hymenidin), together with some of their synthetic analogs, block these channels, featuring high nanomolar to low micromolar potencies [98]. It has been found that clathrodin has high cytotoxicity against the CHO-K1 cells (ED_50_, 1.33 µg mL^−1^), due to the blocking activity against cholinergic receptors, and a modulatory effect on voltage-gated sodium channels [150].

Considering these premises, investigating the effects of brominated chemicals from marine organisms on potassium channels is an intriguing field of study, with potential applications in medication development and discovery. To fully understand the precise mechanisms of action and assess their potential for treatment in a range of illnesses and ailments, more research is necessary.

### 3.2. Secondary Metabolites

**Figure 22:**
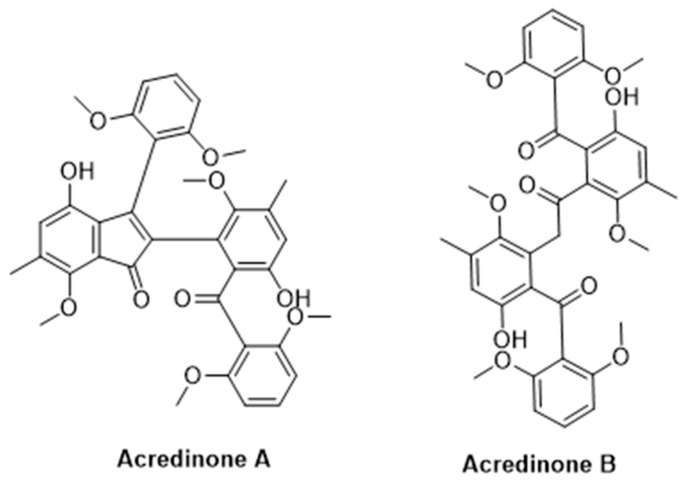
Structure of Acredinones A and B.

Secondary metabolites of the sponge-derived fungus, Acremonium sp. Fungus F9A015, were collected in August 2010 from Gageo Island near the southwest sea of Korea [151]. Two of these metabolites, Acredinones A and B (Figure 22), inhibited the K_v_ channel in INS-1 cells, with IC50 values of 0.59 and 1.0 μM, respectively. The authors of this study assumed the K_v_ channel in INS-1 cells to be K_v_2.1, and therefore suggested the acredinones to be K_v_2.1 inhibitors. They did not directly test these toxins on the K_v_2.1 channels. The K_v_1.7 channels also play a role in INS-1 cells and could be a target for the acredinones. Studies of acredinones on cloned K_v_2.1 and K_v_1.7 channels are warranted [152].

## 4. Additional Non-Peptide K^+^ Channels Modulators 

### 4.1. Gambierol 

**Figure 23:**
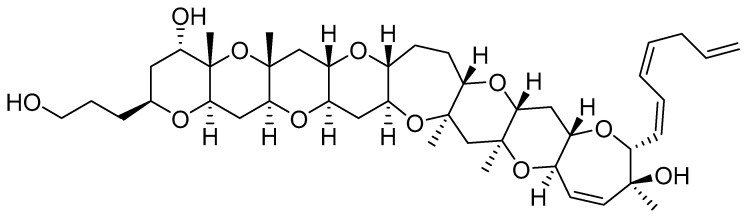
Structure of gambierol toxin, showing the eight polyether rings.

Gambierol, a marine polycyclic ether toxin which is produced by the dinoflagellate *Gambierdiscus toxicus* (Figure 23), belongs to the ciguatoxins family (CTXs). It is composed of eight ether rings, 18 stereocenters, and two pyranyl rings with methyl groups in a 1,3-diaxial orientation. The toxin is reported to accumulate throughout the marine food chain and is believed to be responsible for ciguatera fish poisoning (CFP) [153], characterized by gastrointestinal and neurological symptoms and by hypotension, bradycardia, respiratory difficulties, and paralysis in severe cases [154].

Ciguatoxins have been shown to specifically activate voltage-gated sodium channels, with modest nanomolar affinity [155]. Moreover, some ciguatoxins depolarize the cell membrane, prolong action potential duration, reduce the amplitude of after hyper-polarizations, and lower the threshold for action potential firing in DRG neurons, suggesting potassium channel-blocking activity [156]. Unlike other CTXs, gambierol affects VGSCs with low-efficacy, acting as a partial agonist to stimulate sodium influx in cerebrocortical neurons [157], while blocking several K_v_ channels with IC_50_ values in the nanomolar range (K_v_1.1 = 64 nM; K_v_1.2 = 34.5 nM; K_v_1.3 = 853 nM; K_v_1.4 = 108 nM; K_v_1.5 = 64 nM; K_v_3.1 = 1.2 nM). Notably, gambierol has no effect on K_v_1.6, K_v_2, K_v_4, K_v_11.1/hERG, fly Shaker-IR, and several sodium channels (mammalian Nav1.1–Nav1.8; insect para channel) [154,158]. Gambierol stabilizes the closed state of K_v_3.1 by binding to the lipid-exposed surface of the pore domain [158].

Given the significance of _Kv_1.2 in multiple sclerosis, gambierol may be a promising lead molecule for future research on the application of toxins in the management of pathogenic disorders [159].

### 4.2. Aplysiatoxins

**Figure 24:**
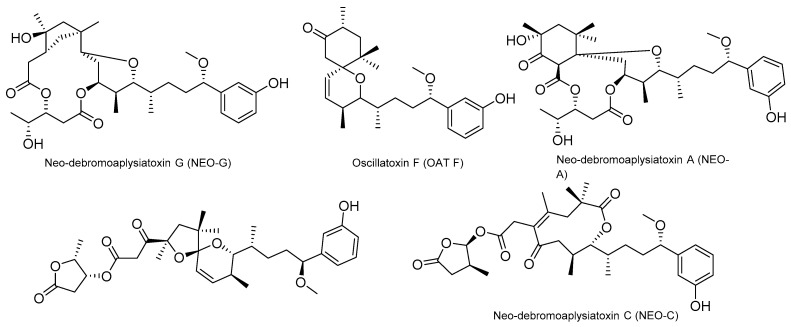
Aplysiatoxins’ molecular structures.

A class of biologically active dermatoxins called Aplysiatoxins (ATXs) were first discovered in the digestive gland of the marine mollusk Stylocheilus longicauda. These toxins are now recognized as metabolic derivatives from cyanobacteria. In fact, ATXs and related analogs, namely oscillatoxins and nhatrangins (Figure 24), are 27 bioactive dermatoxins polyketide compounds that can be isolated from several marine cyanobacteria, endowed with antiproliferative activity, tumor-promoting properties, proinflammatory actions, and antiviral activity [160]. Owing to the existence of certain unstable functional groups, such as ketal and hemiacetal, among others, ATXs were easily rearranged structurally to create a novel structure [161]. According to the structural characteristics, ATXs are divided into three categories: the first group features ABC tricyclic ring systems with carbon numbers of 6/12/6, 6/10/6, or 6/6/6 (e.g., Debromoaplysiatoxins and neo-debromoaplysiatoxins); the second group is characterized by an AB spirobicyclic ring system (e.g., Oscillatoxin D); and the third group includes acyclic structures such as nhatrangins [160]. 

ATXs exhibit remarkable blocking action against K_v_1.5 channels, revealing a possible pivotal target for new treatments of atrial tachyarrhymias with minimal potential for deleterious side effect [57,161]. Researchers proposed two methods for the K_v_1.5 inhibitory mechanism by these different bioactive chemicals, although more research is needed to confirm them. One suggested mechanism involves directly blocking the pore to modulate the ion channel. The alternative theory is that protein kinase C activation indirectly modifies the K_v_1.5 channel [161]. 

## 5. Conclusions

Animal venom contains bioactive chemicals, such as peptide toxins, which target various ion channels (K^+^, Na^+^, Cl^−^, and Ca^2+^). Toxins that target voltage-gated K^+^ (K_v_) channels are common and have been found in the venoms of several animals, including scorpions, sea anemones, snakes, marine and land snails, and spiders. Potassium ion flux through cell membranes is critical for many biological processes and functions, including blood pressure regulation, immunity, neurotransmitter release and nerve conduction, muscle contraction, hormone secretion, intracellular volume regulation, and cell growth and differentiation, to name a few. K^+^ channels are the most diverse family of ion channels, with several subtypes, configurations (two, four, or six transmembrane segments), and functions. Within this family, K^+^ channels, which are selectively permeable to K^+^ ions, are the biggest group and are a requisite for a variety of physiological functions. Due to their functional importance for the electrical excitability of cells, diverse organisms, such as scorpions, anemones, snakes, and snails have developed highly specific toxins targeting the voltage-gated K^+^ channels of their prey. Some of these toxins have been successfully used as probes for scrutinizing the structure and function of ion channels.

This review provides an in-depth investigation of toxin structures and pharmacological selectivities and reveals that toxins with comparable folds can act on multiple subtypes of K_v_ channels, whereas those with unrelated folds may only target a single K_v_ channel. As a result, it seems that a similar spatial distribution of amino acid residues that are essential to the toxin–channel interaction (rather than the type of toxin fold) is required for structurally distinct toxins to interact with a particular K_v_ channel. The diversity of K_v_ channel blockers highlights their potential therapeutic advantage in the treatment of several and specific human difficult-to-treat diseases, particularly autoimmune disorders, inflammatory neuropathies, and cancer. For this reason, marine organisms are a very rich source of molecules not only for studying and deeply investigating the functional aspects of various ion channel isoforms and receptors but also for understanding their role in normal and disease states. In addition, these molecules can help overcome the barriers that exist between the initial drug discovery stage and the clinical use of these toxins, allowing them to be used as treatments.

## Figures and Tables

**Table 1:** Main K^+^ channel targets of conopeptides and relative potencies and/or efficacies.

Conopeptide	Target Channel	Potency or Efficacy	Reference(s)
κ-PVIIA	Fly Shaker	IC_50_= 60 nM	[39]
	hK_v_1.1, hK_v_1.4	IC_50_ = 1 µM	[47]
Mo1659	K^+^ currents in rat DRG	29.6% current amplitude reduction at 200 nM	[48]
κ-BtX	KCa1.1	229% current amplitude increase at 10 nM (EC_50_= 0.7 nM)	[39]
ViTx	rK_v_1.1	IC_50_= 1.59 μM	[49,50]
	rK_v_1.3	IC_50_ = 2.09 μM	[49,50]
κA-SIVA	Fly Shaker	54% current amplitude reduction at 100 nM	[50]
κM-RIIIK	hK_v_1.2	IC_50_ = 352 nM	[51]
	hK_v_1.2/1.7	IC_50_ = 680 nM	[51]
	hK_v_1.2/1.5 and hK_v_1.2/1.1	IC_50_ = 2.76 μM	[51]
	hK_v_1.2/1.1	IC_50_ = 2.80 μM	[51]
	hK_v_1.2/1.6	IC_50_ = 7.70 μM	[51]
	hTSha1	IC_50_ = 20 nM (closed state) 60 nM at 0 mV	[52]
κM-RIIIJ	hK_v_1.2	IC_50_ = 33 nM	[53]
	hK_v_1.2/1.1	IC_50_ = 12 nM	[53]
	hK_v_1.2/1.3	IC_50_ = 165 nM	[53]
	hK_v_1.2/1.4	IC_50_ = 8.13 μM	[53]
	hK_v_1.2/1.5	IC_50_ = 287 nM	[53]
	hK_v_1.2/1.6	IC_50_ = 24 nM	[53]
	hK_v_1.2/1.7	IC_50_ = 370 nM	[53]
Conkunitzin-S1	Fly Shaker	IC_50_ = 502 nM	[54]
	hK_v_1.2	IC_50_ = 3.4 μM	[55]
	hK_v_1.7	IC_50_= 439 nM	[55]
	hK_v_1.2–1.7	IC_50_ = 180 nM	[55]
	hK_v_1.7–1.2	IC_50_ = 390 nM	[55]
	hK_v_1.7–1.2	IC_50_ = 390 nM	[55]
CPY-Pl1	Mammalian K_v_1.2	IC_50_ = 2.0 μM	[56]
	Mammalian K_v_ 1.6	IC_50_ = 170 nM	[56]
CPY-Fe1	Mammalian K_v_ 1.6	IC_50_ = 8.8 nM	[56]
pl14a	Mammalian K_v_1.6	IC_50_ = 1.5 μM	[57]
sr11a	rK_v_1.2	IC_50_ = 640 nM	[58,59]
	hK_v_1.6	IC_50_ = 640 nM	[58,59]

**Table 2:** Main K^+^ channel targets of sea anemones peptides and their relative potencies, efficacies, and affinities.

Sea Anemones Peptide	Target Channel	Potency, Efficacy or Affinity	Reference(s)
BDS-I	hKv3.4	IC_50_ = 47 nM	[87]
	mKv3.1	IC_50_ = 220 nM	[88]
	rKv3.2	48.1% current amplitude inhibition at 500 nM (+40 mV)	[88]
BDS-II	hKv3.4	IC_50_ = 56 nM	[87]
	mKv3.1	IC_50_ = 750 nM	[88]
	rKv3.2	52.5% current amplitude inhibition at 500 nM (+40 mV)	[88]
APETx1	hERG1	IC_50_ = 34 nM	[89]
	hERG3	Equally responsive as hERG1 (determined by Kd)	[90]
BcsTx1	rKv1.1	IC_50_ = 405 nM	[91]
	rKv1.2	IC_50_ = 30 pM	[91]
	hKv1.3	IC_50_ = 74 nM	[91]
	rKv1.6	IC_50_ = 1.31 nM	[91]
	Shaker IR	IC_50_ = 275 nM	[91]
BcsTx2	rKv1.1	IC_50_ = 14.4 nM	[91]
	rKv1.2	IC_50_ = 80.4 nM	[91]
	hKv1.3	IC_50_ = 13.1 nM	[91]
	rKv1.6	IC_50_ = 7.76 nM	[91]
	Shaker IR	IC_50_ = 49.1 nM	[91]
BcsTx3	rKv1.2	IC_50_ = 172.6 nM	[92]
	rKv1.3	IC_50_ = 1.0 μM	[92]
	rKv1.6	IC_50_ = 2.2 μM	[92]
	Shaker IR	IC_50_ = 94.2 nM	[92]
AsKC1	rKv1.2	IC_50_ = 2.8 µM	[93]
AsKC2	rKv1.2	IC_50_ = 1.1 µM	[93]
AsKC3	rKv1.2	IC_50_ = 1.3 µM	[93]
APEKTx1	rKv1.1	IC_50_ = 0.9 nM	[94]
SHTX-III	rKv1	Ki = 650 nM	[95]
AbeTx1	rKv1.1	IC_50_ = 672 nM	[96]
	rKv1.2	IC_50_ = 167 nM	[96]
	rKv1.6	IC_50_ = 116 nM	[96]
Ate1a	rKv1.1	IC_50_ = 33 nM	[95]
	rKv1.2	IC_50_ = 12 nM	[95]
	hKv1.3	IC_50_ = 3.0 μM	[95]
	rKv1.6	IC_50_ = 191 nM	[95]
BgK	rKv1.1	Ki = 34 pM	[97]
	rKv1.2	Ki = 66 pM	[97]
	rKv1.3	Ki = 777 pM	[97]
	rKv1.6	Ki = 13 pM	[97]
ShK	mKv1.1	Ki = 16 pM	[98]
	rKv1.2	Ki = 9 nM	[98]
	mKv1.3	Ki = 11 pM	[98]
	hKv1.6	Ki = 165 pM	[98]
	mKv1.7	Ki = 11.5 nM	[98]
	hKCa4	Ki = 28 nM	[98]
HmK	hKv1.2	Ki = 2.5 nM	[99]
	hKv1.3	Ki = 3.1 nM	[99]
	Fly Shaker	Ki = 1.0 nM	[99]
AsKs	rKv1.2	IC_50_ = 140 nM	[93]
AeK	rKv1	IC_50_ = 22 nM	[100]
AETXk	hKv1.2	Ki = 2.2 µM	[99]
	hKv1.3	Ki = 1.3 µM	[99]
	Fly Shaker	Ki = 445 nM	[99]
